# Evaluation of Library Preparation Workflows and Applications to Different Sample Types Using the PowerSeq^®^ 46GY System with Massively Parallel Sequencing

**DOI:** 10.3390/genes14050977

**Published:** 2023-04-26

**Authors:** Kyleen Elwick, Patrick Rydzak, James M. Robertson

**Affiliations:** 1 Visiting Scientist Program, Research & Support Unit, Laboratory Division, Federal Bureau of Investigation, 2501 Investigation Parkway, Quantico, VA 22135, USA; patrick.m.rydzak@nasa.gov; 2 Research & Support Unit, Laboratory Division, Federal Bureau of Investigation, Quantico, VA 22135, USA; jmrobertson@fbi.gov

**Keywords:** massively parallel sequencing (MPS), next-generation sequencing (NGS), PowerSeq^®^ 46GY System, STRs, library preparation, libraries, purification beads

## Abstract

This project evaluated the prototype PowerSeq^®^ 46GY System using donor DNA and casework-type samples. The goal of this study was to determine whether modifications to the manufacturer’s protocol could increase read coverage and improve sample results. Buccal and casework-type libraries were prepared using the TruSeq^®^ DNA PCR-Free HT kit or the KAPA HyperPrep kit. Both kits were evaluated unmodified, and by substituting AMPure^®^ XP beads for the beads of the most optimal kit. Two qPCR kits, the PowerSeq^®^ Quant MS System and KAPA Library Quantification Kit, were also evaluated along with a KAPA size-adjustment workbook, which was compared as a third quantification method. Libraries were sequenced using the MiSeq^®^ FGx and data were analyzed with STRait Razor. Results suggested that all three quantification methods overestimated library concentration, but the PowerSeq kit was most accurate. Samples prepared with the TruSeq library kit provided the highest coverage and the fewest instances of dropout and below-threshold alleles compared with the KAPA kit. Additionally, all bone and hair samples demonstrated full profile completeness, with bone samples yielding a higher average coverage than hair samples. Overall, our study demonstrated that the 46GY manufacturer’s protocol produced the best quality results compared to alternative library preparation options.

## 1. Introduction

Short tandem repeats (STRs) have been used for human identification in forensic science for over 20 years [1,2,3] and are ideal for forensic casework because of the high power of discrimination they provide between individuals. Additionally, the Combined DNA Index System (CODIS) contains over 20 million STR reference profiles [4], and the system is nationally recognized. In 2011, a new technique referred to as next-generation sequencing (NGS), massively parallel sequencing (MPS), or second-generation sequencing (SGS) was first used for forensic applications to analyze multiple targeted STR loci simultaneously [5,6]. Fordyce et al. reported the use of MPS to characterize ten individuals with five STR loci [5]. They found that sequencing STRs, rather than using capillary electrophoresis (CE) or fragment analysis, provided better resolution of the STRs, e.g., sequence variation. In 2012, all 13 core CODIS STR loci and the amelogenin sex marker were successfully sequenced in individuals and mixtures [6]. Since the discovery of MPS for use with forensic applications, adoption has been slow due to the rigorous testing and validation needed to incorporate new technology into forensic laboratories. 

In the past 10 years, substantial research and development has provided the forensic community with numerous panel options using markers for MPS analysis of STRs [7,8], single nucleotide polymorphisms (SNPs) [9,10], microhaplotypes [11], and mitochondrial DNA [12,13]. Although STR analysis via CE is still the standard for forensic casework analysis, several studies have proposed MPS as an alternative because it offers a potential solution to many common challenges of STR analysis, including large multiplexes and sample mixtures [14], the increased genotyping success of degraded DNA and cost reduction compared to CE [15], and the increased success of challenging sample types [16]. Additionally, challenges still exist for the implementation of MPS into forensic casework, which is one reason for the study presented here.

To perform MPS analysis of a sample, a library of DNA fragments is constructed and sequenced. The forensic workflow of DNA library preparation prior to MPS includes several steps such as DNA extraction, PCR amplification of target regions, DNA purification, adaptor ligation, and library quantification. Numerous studies have focused on modifications to each of these steps for applications in human identification using various chemistries and across multiple sequencing platforms for autosomal analysis [17,18,19]. Additionally, many laboratories have already implemented MPS technology for use with either criminal casework or human identification [10,20,21,22,23,24,25,26,27]. 

In response to the increased interest in MPS, several manufacturers provide various kits. Some are amplification kits that require the purchase of a separate library preparation kit (PowerSeq^®^ 46GY System (Promega Corporation, Madison, WI, USA)), while others contain all the reagents necessary for DNA amplification and library preparation: Precision ID GlobalFiler™ NGS STR Panel v2 (Thermo Fisher Scientific, Waltham, MA, USA), and the ForenSeq DNA Signature Prep Kit (Verogen, Inc., San Diego, CA, USA). These products provide the user with a complete primer panel capable of amplifying makers commonly used in human identification. Several publications have reported on the use of the ForenSeq DNA Signature Prep Kit [18,28,29] and Precision ID GlobalFiler NGS STR Panel [30,31,32], among other panels. The PowerSeq^®^ 46GY System (previously the PowerSeq™ Auto/Y System) was evaluated for use with human population identification [7,33,34,35,36], STR analysis of forensic-type samples [37], standard reference materials [38], and modifications of individual steps within the PowerSeq^®^ workflow [37,39]. However, there are no published works on the optimization of the entire workflow of the PowerSeq^®^ 46GY System. Before any protocol is used for forensic casework, there is a need to assess the downstream effects of potential protocol-enhancing modifications to the manufacturers’ suggested protocols to ascertain if modifications will increase the quantity and quality of DNA libraries or affect the concordance of identification results.

The goal of this study was to identify potential enhancements to the existing manufacturer protocol for the PowerSeq^®^ 46GY System and TruSeq workflow, then assess the effects of these modifications on casework-type samples through sequencing. To accomplish this, library preparation was performed using two kits, libraries were purified using three solid-phase reversible immobilization (SPRI) beads, and libraries were quantified with two kits. Each library was constructed using donor buccal samples, followed by sequencing on the Illumina MiSeq FGx™ (Illumina, San Diego, CA, USA). The most efficient protocol was selected based on sequencing data metrics obtained from buccal DNA-based libraries and applied to forensic-type bone and hair samples.

## 2. Materials and Methods

### 2.1. DNA Samples

Two known buccal sources, one male and one female, were used as standards to test library preparation modifications. Single hairs were collected from four individuals, and bones from six individuals were provided by the Southeast Texas Applied Forensic Science Facility (STAFS) in Huntsville, TX, USA. Human hair and bone samples were used as casework-type samples to assess the downstream impact of library preparation modifications on sequencing results. Information regarding the bone and hair samples may be found in Supplemental Table S1. Buccal and hair samples were collected with informed consent according to Internal Review Board (IRB) guidelines. 

### 2.2. DNA Extraction and Quantification

#### 2.2.1. Buccal Swab Extraction

Buccal swabs were extracted using a semi-automated extraction protocol [40]. The swab heads were placed in Investigator Lyse&Spin baskets (QIAGEN, Germantown, MD, USA) with 450 µL of lysis buffer (Buffer G2, Proteinase K (PK, 20 mg/mL) and dithiothreitol (DTT, 1 M)) and lysed for one hour at 56 °C with gentle agitation (~200 rpm). The purification of the lysates was performed with the EZ1 Advanced XL (QIAGEN, Hilden, Germany) using the Large Volume Protocol and eluting the DNA in 50 µL of nuclease-free water (hereafter termed ‘water’). 

#### 2.2.2. Hair Extraction

Prior to use, hairs were stored at room temperature in individual, sealed and sterile plastic bags. Hair samples were prepared and extracted using a manual extraction and purification method [41]. Hairs were cut approximately 2 cm from the root and individually placed in separate 1.5 mL tubes. Samples were purified using the PrepFiler^®^ DNA Extraction Kit (Life Technologies, Carlsbad, CA, USA) following [41] and eluted in 65 µL of Elution Buffer. 

#### 2.2.3. Bone Extraction

Bone powder (approximately 0.2 g) was weighed out (in triplicate) for each sample and placed in separate 15 mL conical tubes. To each tube, 3 mL demineralization (Demin) Buffer (0.5 M EDTA, 1% N-lauroylsarcosine sodium salt at pH 8) and 200 µL of PK were added. The tubes were gently vortexed, then incubated in a thermomixer with agitation at 900 rpm for 24 h at 56 °C. Extraction was completed following [42], using an Amicon Ultra-4 Centrifugal Filter Units with a 30 KDa membrane (MilliporeSigma, St. Louis, MO, USA) instead of a Vivacon^®^ 2 concentrator. Samples were eluted in 50 µL water according to [42]. All bone samples were extracted in triplicate and combined for a larger volume. Reagent blanks were included for each extraction. Extracts were stored at −20 °C until use. 

#### 2.2.4. DNA Quantification

All extracts were quantified using Quantifiler™ Trio DNA Quantification Kit (Thermo Fisher Scientific) on a 7500 Real-Time PCR System (Thermo Fisher Scientific) as per the manufacturer’s protocol [43]. Data were analyzed using HID Real-Time PCR Analysis Software v1.2. 

### 2.3. PowerSeq^®^ Amplification and Library Preparation

For the first three studies, male and female buccal extracts were amplified using 30 PCR cycles in duplicate at five DNA inputs (1 ng, 0.5 ng, 0.25 ng, 0.125 ng, 0.063 ng), and a negative and positive control were included with each experiment (*n* = 22). For the final study, six bone and four hair extracts were amplified in duplicate in addition to a bone and hair reagent blank and a positive and negative control (*n* = 24). Hair and bone extracts were amplified with 0.5 ng DNA or the maximum sample volume (15 µL) if DNA was less than 0.033 ng/µL, as per the manufacturer’s recommendations [44]. For positive control samples, 0.5 ng of 2800 M Control DNA (Promega) was used. Note that the Promega Technical Manual has since been updated to target 1 ng of DNA input and 29 PCR cycles [45]. 

#### 2.3.1. TruSeq DNA PCR-Free HT Library Preparation with SPBs

Sample extracts (15 µL) were amplified using 5 µL PowerSeq^®^ 5X Master Mix and 5 µL PowerSeq^®^ 46GY 5X Primer Pair Mix with the following thermal cycling conditions: denaturation for 1 min at 96 °C, followed by 30 cycles of 96 °C for 5 s, 60 °C for 35 s, and 72 °C for 5 s, with a final extension for 2 min at 60 °C. Following amplification, libraries were prepared following the protocol described in the PowerSeq^®^ 46GY System Technical Manual [44] using the TruSeq DNA PCR-Free HT Library Preparation Kit with Sample Purification Beads (SPBs, Illumina). Quantification of purified amplification products was performed using the Qubit™ dsDNA High Sensitivity Assay Kit (Thermo Fisher Scientific) on the Qubit 3.0 Fluorometer (Thermo Fisher Scientific) or with the Agilent 2100 Bioanalyzer System (Agilent Technologies Inc., Fairmont, WV, USA) using the Agilent High Sensitivity DNA Kit (Agilent). 

#### 2.3.2. KAPA HyperPrep Library Preparation with KPBs

The KAPA HyperPrep Kit (Roche Sequencing Solutions, Pleasanton, CA, USA) protocol recommends fragmentation of the DNA, followed by end repair and A-tailing, adapter ligation, post-ligation cleanup, library amplification, and post-amplification cleanup prior to sequencing. A modified version of the KAPA HyperPrep Kit protocol was adopted to better suit the library preparation workflow of the 46GY Panel and fragmentation was omitted. Buccal sample extracts (15 µL) were amplified using 25 µL KAPA HiFi HotStart ReadyMix (2×) and 10 µL PowerSeq^®^ 46GY 5× Primer Pair Mix with the following thermal cycling conditions: 1 min denaturation at 98 °C, followed by 30 cycles of 5 s at 98 °C, 35 s at 60 °C, and 5 s at 72 °C, and finally, a 1 min final extension at 72 °C. Post amplification required a 1× bead-based cleanup using 50 µL KAPA Pure Beads (KPBs, Roche Sequencing Solutions). The plate was incubated for 10 min at room temperature and then placed on a magnetic stand until the liquid was clear (~10 min). The supernatant was discarded and 200 µL 80% ethanol was added, followed by a 30 s incubation before removal, ensuring the beads remained attached to the tube wall. The ethanol cleanup was performed once more for a total of two cleanups. The beads were dried for 3 to 5 min at room temperature while residual ethanol evaporated. The plate was removed from the magnetic stand and the beads were resuspended in 55 µL water. The purified amplification product (50 µL) was transferred to a new plate. End repair and A-tailing were completed in a single step. Clean, amplified product (50 µL) was combined with 7 µL End Repair and A-Tailing Buffer and 3 µL End Repair and A-Tailing Enzyme Mix. The samples were incubated for 30 min at 20 °C followed by 30 min at 65 °C. Samples were quantified using the Qubit™ dsDNA High Sensitivity Assay Kit with the Qubit 3.0 Fluorometer to correctly dilute adapter stocks from the KAPA Dual Indexed Adapter Kit (Roche). Based on the quantities of the end repaired and A-tailed libraries, adapters were diluted 1:10, 1:20, or 1:40. For adapter ligation, 60 µL end repair and A-tailing reaction product was combined with 5 µL adapter stock, 5 µL water, 30 µL Ligation Buffer, and 10 µL DNA Ligase, which was incubated for 15 min at 20 °C. Next, a post-ligation cleanup was performed using 88 µL (0.8× ratio) KPBs with two ethanol cleanup steps, as described previously. The libraries were eluted in 55 µL water and 50 µL was transferred to a new plate for double-sided size selection. An aliquot (35 µL, 0.7× ratio) of KPBs was added to 50 µL library and incubated for 10 min at room temperature. The plate was placed on a magnetic stand and 80 µL supernatant was transferred to a new plate. A small aliquot (10 µL) KPBs was added to 80 µL supernatant from the first size selection. Two ethanol cleanup steps were performed as described previously. Samples were eluted in 25 µL Tris-HCl (pH 8.0–8.5) and 20 µL final product was transferred to a new plate. 

#### 2.3.3. TruSeq Library Preparation with AMPure XP Bead Purification

This study was carried out exactly as the first TruSeq study except for the use of AMPure XP beads (Agencourt Bioscience Corporation, Beverly, MA, USA) in place of the SPBs included in the TruSeq DNA PCR-Free HT Library Preparation Kit. 

#### 2.3.4. Casework-Type Samples with Enhanced Library Preparation Method

The library preparation protocol chosen for casework-type samples was based on quantification and sequencing results of buccal samples from the previous three studies. Based on these results, the TruSeq DNA PCR-Free HT Library Preparation Kit was chosen along with Illumina SPBs and the protocol described in 2.3.1. 

### 2.4. Library Quantification, Normalization, and Illumina Sequencing

#### 2.4.1. Library Quantification

Libraries were quantified with two different MPS library quantification kits: the PowerSeq^®^ Quant MS System (Promega) [46] and the KAPA Library Quantification Kit for Illumina^®^ Platforms (Roche) [47]. Libraries were quantified in duplicate, using the respective MPS library quantification kits and protocols [46,47], on the QuantStudio™ 5 Real-Time PCR System (Applied Biosystems). Data were analyzed using QuantStudio™ Design & Analysis Software v1.4 and library concentrations were adjusted based on the dilution factor for each kit. Additionally, Roche Sequencing Solutions provided an Excel workbook (KAPA Library Quantification Data Analysis Template) that calculated the concentration of the undiluted library by multiplying the calculated average concentration (in pM) by the equation below [47] for the size-adjusted concentration (in pM) and then by the dilution factor:

(Size of DNA Standard in bp (452))/(Average fragment length of library in bp).

#### 2.4.2. Library Normalization and Quality Check

Libraries were diluted to 4 nM using Resuspension Buffer, and 5 µL of each dilution was pooled in a single tube. The diluted library pool and select samples were quality checked to determine the concentration of the sample pool after library dilution using the Agilent 2100 Bioanalyzer via the Agilent High Sensitivity DNA Kit. To denature the normalized libraries, an aliquot of the ~0.5 nM–4 nM pooled libraries (5 µL (4 nM)–40 µL (0.5 nM)) was combined with an equal volume of 0.2 N NaOH and incubated at room temperature for 5 min. Following denaturation, an equal volume (5 µL–40 µL) of 200 mM Tris-HCl, pH 7, was added to balance the pH of the solution for sequencing. Lastly, a Hybridization Buffer (880 µL–985 µL, depending on the other components added previously) was added to the tube containing the denatured library for a final library concentration of 20 pM. A PhiX Control (Illumina) was diluted and denatured by combining 2 µL PhiX Control (10 nM), 3 µL Resuspension Buffer, and 5 µL 0.2 M NaOH to incubate at room temperature for 5 min. Following incubation, 990 µL Hybridization Buffer was added for a final concentration of 20 pM. Finally, the sequencing dilution was created by combining 195 µL Hybridization Buffer, 365 µL of the 20 pM pooled and denatured libraries, and 40 µL of the 20 pM denatured PhiX Control. 

#### 2.4.3. Illumina Sequencing

Sequencing was performed on an Illumina MiSeq FGx™ using a standard flow cell and 600-cycle v3 kit with 2 × 300 bp reads and 2 × 8 cycles for sample indices. For each of the first three studies (Section 2.3.1, Section 2.3.2 and Section 2.3.3), 22 PowerSeq^®^ normalized sample libraries were pooled for sequencing. For the casework study (Section 2.3.4), 24 PowerSeq^®^ normalized sample libraries were pooled for sequencing. 

### 2.5. Data Analysis

Sequencing results and quality metrics were observed using Sequence Analysis Viewer software (Illumina) [48]. FASTQ files were analyzed using STRait Razor Online (SRO) [49] and sequences were aligned to human genome assembly GRCh38. An analytical threshold of 50 reads was applied to STR genotypes according to SWGDAM guidelines [50]. An interpretation threshold of 500 reads for homozygotes and 100 reads for heterozygotes was applied to autosomal loci according to Hölzl-Müller et al. [51]. Additionally, an interpretation threshold of 100 reads was also applied to Y-STRs according to Moon et al. [52]. Reads above 50× coverage but below the interpretation threshold were considered “Below-Threshold” (BT). Loci below the analytical threshold or with no coverage were considered “Dropout” (DO). Heterozygote imbalance was called for ratios < 0.50, a threshold used by Zeng et al. [37]. Furthermore, locus DYS389I is left out of the default STRait Razor analysis for the 46GY panel and therefore may not be optimally represented in the analyses of this study. This study assessed profile completeness (%), coverage range, average coverage, heterozygote balance (HB: the coverage of the lesser allele divided by the coverage of the larger sister allele), below-threshold alleles, drop-in alleles, and dropout. Data were compared to GlobalFiler CE reference profiles on file for buccal and hair samples, and STAFS provided GlobalFiler references for the bone samples. MPS references were produced by consensus from the replicates. Statistical significance was ascertained using an F-test Two-Sample for Variances followed by a Two-Sample, two tailed *t*-Test assuming equal (or unequal) variances or ANOVA using α 0.05. All statistical analyses were performed using IBM SPSS Statistics Version 29.0 (IBM Corporation, Armonk, NY, USA).

## 3. Results and Discussion

### 3.1. TruSeq DNA PCR-Free HT Library Preparation

Library concentrations ranged from 15 nM to 272 nM (averages 34 nM (63 pg)–188 nM (1 ng)) when using the PowerSeq^®^ Quant MS System (PowerSeq^®^), 19 nM to 1151 nM (averages 43 nM (63 pg)–506 nM (1 ng)) when using the KAPA quantification kit (KAPA) prior to size adjustment, and from 25 nM to 1530 nM (averages 58 nM (63 pg)–669 nM (1 ng)) when using KAPA’s data analysis template (fragment length adjustment) (Supplemental Figure S1 and Supplemental Table S2). The KAPA size-adjustment method reported concentrations approximately 1.3× higher than the concentrations calculated prior to size adjustment and approximately 1.7×–5.6× higher than concentrations calculated using the PowerSeq^®^ kit. The KAPA size-adjusted concentrations were higher than most PowerSeq^®^ and all non-size-adjusted KAPA concentrations (Supplemental Table S2). When samples were normalized to 4 nM and pooled, the final sample concentration was quantified via the DNA High Sensitivity Kit using the 2100 Bioanalyzer. The pooled libraries normalized by each library quantification method resulted in final library concentrations below the 4 nM target, meaning that libraries were overestimated by qPCR and thus overdiluted. However, PowerSeq-reported library concentrations were the most accurate, relative to final quantification with the bioanalyzer, and could be adjusted for sequencing during library denaturation and dilution. The reason the PowerSeq^®^ and KAPA kits resulted in largely different values, considering both chemistries use similar quantitative principles by measuring the number of adapter-ligated molecules available for sequencing, is uncertain. However, it may be due to the sensitivity of the KAPA kit to concatemers causing an artificially high quantification value per sample (personal communication—KAPA technical support specialist). 

**Figure 1:**
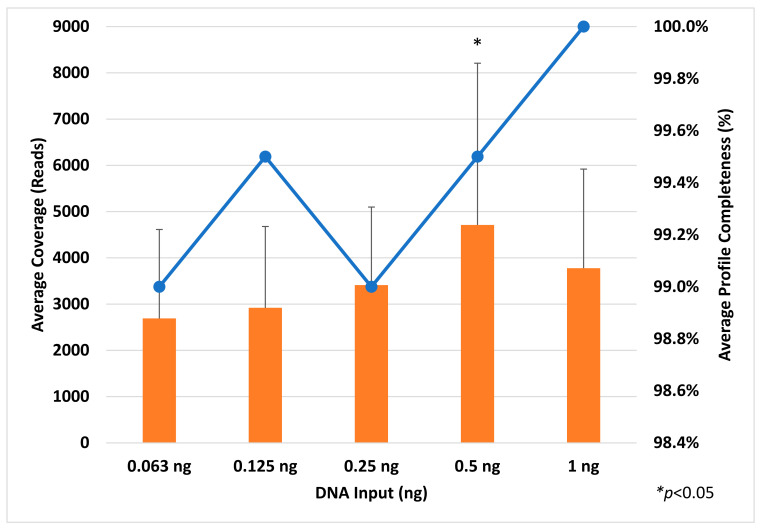
Average coverage on the *y* axis (orange bars) and average profile completeness (blue line) on the secondary *y* axis for each DNA input (0.063–1 ng) amount on the *x* axis. These results represent the TruSeq library preparation kit using SPBs. Error bars = ±SD.

**Figure 2:**
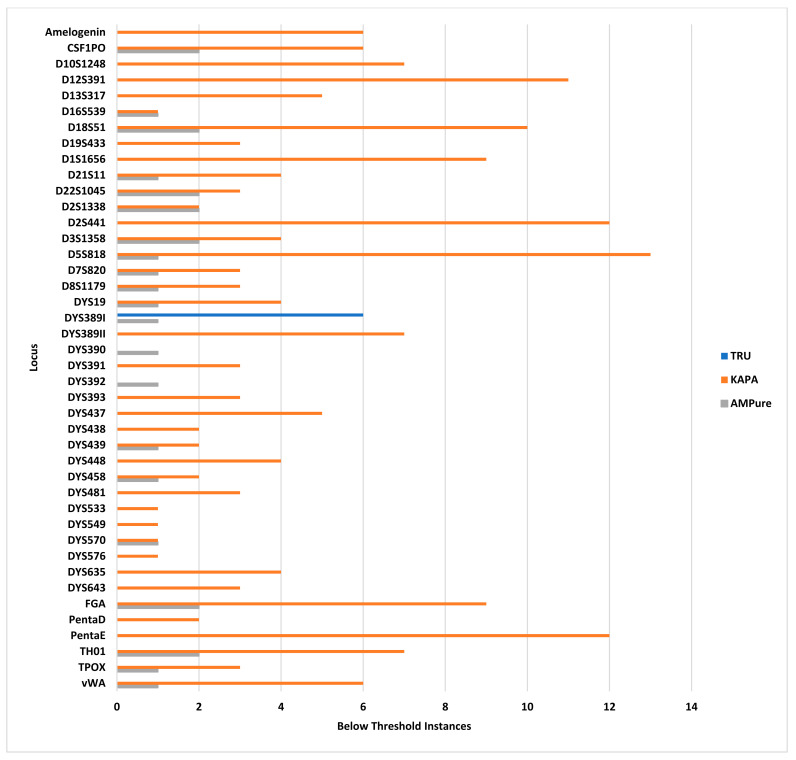
Instances of below threshold alleles (*x* axis) for each of the three library preparation/purification methods per locus (*y* axis): TruSeq library preparation with SPBs (blue), KAPA library preparation with KPBs (orange), and TruSeq library preparation with AMPure beads (gray).

**Figure 3:**
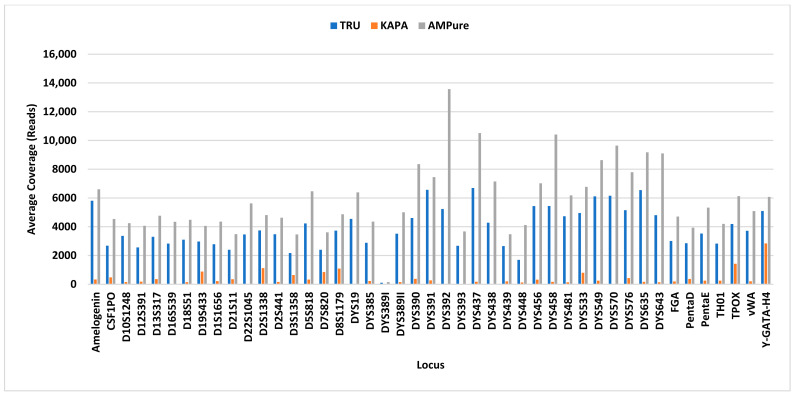
Average coverage (*y* axis) per locus (*x* axis) for each library preparation/purification method: TruSeq library preparation with SPBs (blue), KAPA library preparation with KPBs (orange), and TruSeq library preparation with AMPure beads (gray).

Profile completeness using the TruSeq library preparation kit ranged from 98.5% to 100% (Figure 1), with a single allele (DYS389I) below the interpretation threshold of 100 reads for six out of ten male samples (Figure 2, single blue bar). With future iterations of STRait Razor, utilizing each target of the 46GY panel, full profiles will likely be achievable at 63 pg DNA inputs and possibly lower. Additionally, all loci amplified (Figure 3, blue bars) and demonstrated high coverage at all five DNA inputs (0.063 ng–1 ng); however, the 0.5 ng input demonstrated significantly higher (*p* < 0.001) coverage than all other inputs except for 1 ng (Figure 1). This result was expected, as the PowerSeq^®^ 46GY Technical Manual [44] had been optimized for an input of 0.5 ng of DNA. Coverage ranged from 101× (0.125 ng) to 28,079× (0.5 ng), with average coverage between 2690× (0.063 ng) and 4712× (0.5 ng) (Figure 1 and Figure 3, blue bars). Using the TruSeq library preparation, the locus with the highest average coverage was DYS437, whereas Moura-Neto et al. [36] found that DYS439 demonstrated the highest coverage throughout all samples. However, they also found that DYS389I/II (combined data in their study) and DYS448 demonstrated the lowest read depth. We also observed this trend for DYS389I and DYS448, but not for DYS389II (Figure 3). The heterozygote balance ranged from 0.41 (63 pg) to 0.99 (0.25, 0.5, and 1 ng). Additionally, the average heterozygote balance was >0.70 for all DNA input amounts and increased as the DNA concentration increased, ranging from 0.70 at 63 pg to 0.87 at 1 ng (Supplemental Figure S2). Moura-Neto et al. [36] also observed heterozygosity of the autosomal loci, ranging from 0.70 to 0.85. In this study, all heterozygous loci had an average HB of ≥0.73, with the lowest being FGA and the highest being D5S818 with a 0.91 ratio, indicating no heterozygote imbalance (<0.50) (Supplemental Figure S3). 

Furthermore, there were no instances of dropout or drop-in using the TruSeq library preparation kit with SPBs. All data were concordant with the reference samples, and negative controls were free of contamination. Of note, at the time this work was completed, the suggested DNA input was 0.5 ng using 30 PCR cycles. However, the technical manual, revised 3/22, suggests a DNA input amount of 1 ng using 29 PCR cycles [45]. 

### 3.2. KAPA HyperPrep Library Preparation

Library concentrations ranged from 0.1 nM to 13 nM (averages 0.6 nM (63 pg)–8 nM (0.5 ng)) using the PowerSeq^®^ quantification kit, 0.6 nM to 59 nM (averages 2 nM (63 pg)–29 nM (0.5 ng)) for the KAPA quantification kit, and from 1 nM to 78 nM (averages 3 nM (63 pg)–39 nM (0.5 ng)) using KAPA’s data analysis template (Supplemental Figure S4 and Supplemental Table S3). The KAPA size-adjustment method reported concentrations approximately 1.5× higher than concentrations calculated prior to size adjustment, and approximately 6×–10× higher than concentrations calculated using the PowerSeq^®^ kit. Similar trends were observed when samples were prepared with the TruSeq library preparation kit. 

**Figure 4:**
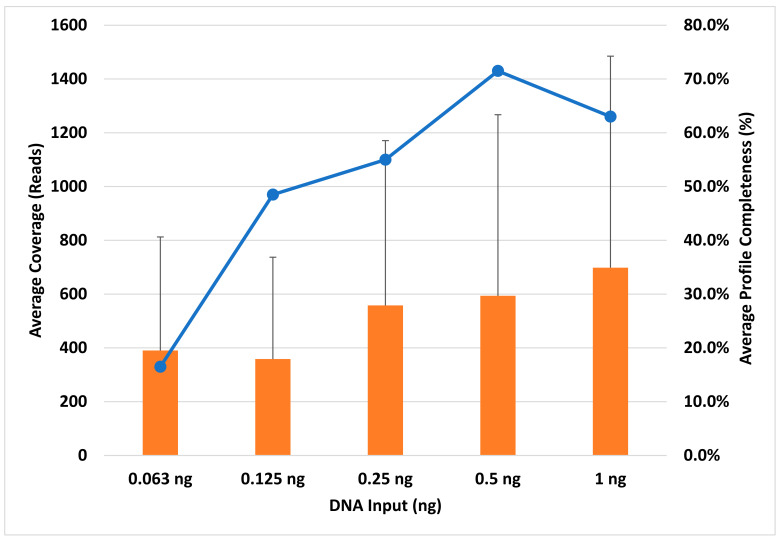
Average coverage on the *y* axis (orange bars) and average profile completeness (blue line) on the secondary *y* axis for each DNA input (0.063–1 ng) amount on the *x* axis. These results represent the KAPA library preparation kit using KPBs. Error bars = ±SD.

The profile completeness ranged from 0% to 92.7%, with an average profile completeness of 17% to 72% (Figure 4). Unlike the TruSeq kit, coverage generally increased as the DNA concentration increased. Overall, for the KAPA HyperPrep kit, coverage was relatively low for all DNA inputs and the amplification of the loci was highly variable, resulting in inconsistent locus coverage across all samples (Supplemental Figure S5). Locus coverage ranged from 0× (dropout) to 5356× (1 ng), with the average coverage between 358× (0.125 ng) and 698× (1 ng) (Figure 4), which is approximately 5×–8× lower than the average coverage obtained using the TruSeq kit and SPBs. Of the loci that amplified using the KAPA kit, Y-GATA-H4 yielded the highest average coverage and DYS448 yielded the lowest average coverage, the latter being similarly observed in Moura-Neto et al. [36] and Silva et al. [34] (Figure 3, orange bars). Excluding dropout (heterozygote balance of zero), heterozygote balance otherwise ranged from 0.40 (0.125 ng) to 1 (1 ng). Average HB was ≥0.14 for all DNA inputs, generally increasing as the DNA concentration increased, ranging from 0.14 (63 pg) to 0.74 (0.5 ng) (Supplemental Figure S2). DNA inputs of 63 pg and 0.125 ng resulted in <0.50 average heterozygote balance, i.e., imbalance, although Zeng et al. [37] only observed more heterozygote imbalance at 62 pg and below. Additionally, two loci (D16S539 and D22S1045) exhibited complete dropout in all samples, as discussed below. Barring these two loci, all heterozygous loci had an average HB ≥ 0.11, with D18S51 being the lowest and D19S433 being the highest at 0.78 (Supplemental Figure S3). In addition, seven of the twenty-one remaining loci without dropout (33%) demonstrated heterozygote imbalance, including D10S1248, D12S391, D18S51, D1S1656, D2S441, FGA, and PentaE (Supplemental Figure S3). Zeng et al. [37] noted that this imbalance is likely due to alleles having a greater size differential at certain loci, including D2S1338 and PentaE. However, this could depend on the genotype specific allele ranges, which vary based on the donor. We observed this trend in Penta E, but not in D2S1338. 

**Figure 5:**
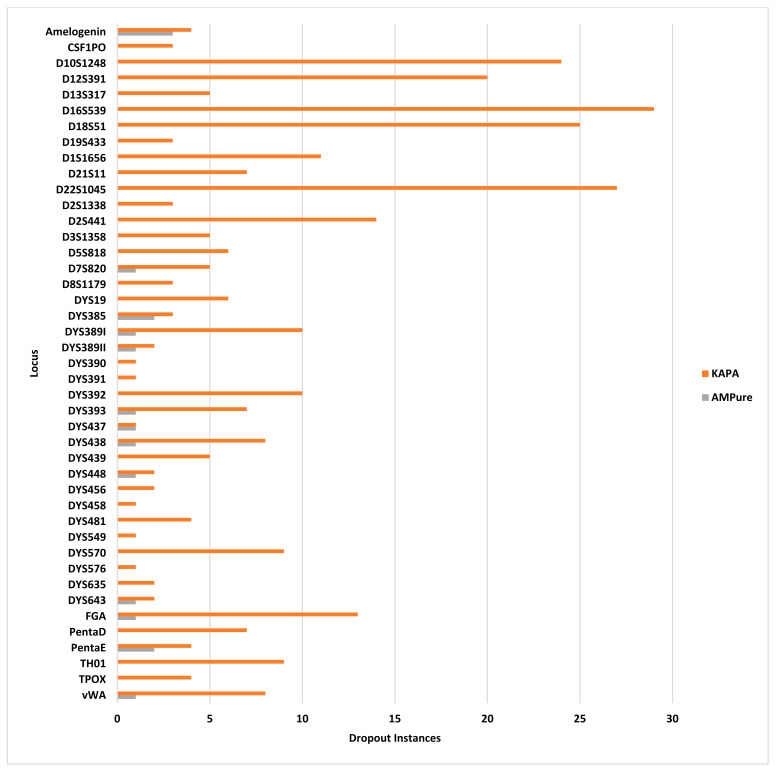
Instances of allelic dropout (*x* axis) per locus (*y* axis) for two of the three library preparation/purification methods: KAPA library preparation with KPBs (orange) and TruSeq library preparation with AMPure beads (gray). TruSeq library preparation with SPBs had no instances of allelic dropout.

Coverage below 50× indicated dropout, and occurred in every sample and from many loci, totaling 317 instances with the HyperPrep kit, the most dropout of the three library preparation methods. For the female samples, there were 154 instances of dropout including two entire samples, which failed to amplify. For the male samples, there were 163 instances of dropout (Figure 5, orange bars); however, unlike the female samples, every sample amplified. A total of 43 (93.5%) of 46 loci resulted in dropout instances, including 29 at D16S539, 27 at D22S1045, 25 at D18S51, 24 at D10S1248, 20 at D12S391, and 14 or fewer for the remaining loci. Two Y-STRs (DYS392 and DYS389I) dropped out in every male sample (Figure 5). No drop-in alleles were observed when the samples were prepared using the KAPA kit. 

Additionally, the KAPA kit produced the most instances of below-threshold alleles, with a total of 187 instances, occurring in every sample (Figure 2, orange bars). Below-threshold alleles were observed in 39 of the 46 loci (84.8%), with 13 instances in D5S818, 12 instances in PentaE and D2S441, 11 instances in D12S391, and 10 instances or fewer in the remaining loci (Figure 2). All data were concordant with the reference samples, and negative controls were free of contamination. 

Riman et al. [53] compared the TruSeq HT Library Preparation Kit with the KAPA HyperPrep Kit and found that the KAPA-prepared libraries exhibited higher yields of adapter-ligated libraries at all DNA input amounts, while our study revealed opposite findings (Supplemental Tables S2 and S3). However, in our study, the KAPA protocol was not optimized for the 46GY panel like the TruSeq protocol. Unlike TruSeq, modifications were required for the KAPA protocol to be compatible with the 46GY panel and a forensic workflow. These modifications included changes to the amplification temperatures and cycle number, adapter concentrations, and bead-to-template ratios. The optimization issues may have been the cause of KAPA’s noticeably lower library yield, profile completeness, and average coverage. In this study, the minimum modifications possible were made to the KAPA protocol to make it compatible with the 46GY workflow. It is possible that with further alterations and optimization, the KAPA kit may generate higher average coverage with the 46GY panel than that reported here. 

### 3.3. TruSeq Library Preparation with AMPure XP Bead Purification

Library concentrations ranged from 6 nM to 411 nM (averages 14 nM (63 pg)–313 nM (1 ng)) using the PowerSeq^®^ kit, 32 nM to 2004 nM (averages 67 nM (63 pg)–1432 nM (1 ng)) using the KAPA kit, and 43 nM to 2661 nM (averages 89 nM (63 pg)–1903 nM (1 ng)) using KAPA’s data analysis template (Supplemental Figure S6 and Supplemental Table S4). KAPA size-adjusted concentrations were approximately 1.3× higher than concentrations calculated prior to size-adjustment, and approximately 6.3× higher than concentrations calculated using the PowerSeq^®^ kit, which was similar to what was observed in the two previous experiments when libraries were prepared via the TruSeq and KAPA library preparation kits. 

**Figure 6:**
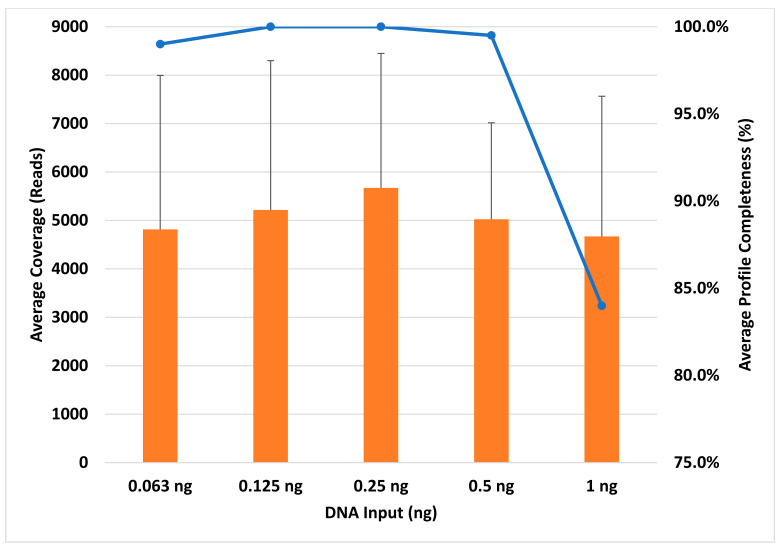
Average coverage on the *y* axis (orange bars) and average profile completeness (blue line) on the secondary *y* axis for each DNA input (0.063–1 ng) amount on the *x* axis. These results represent the TruSeq library preparation kit using AMPure XP beads. Error bars = ±SD.

**Figure 7:**
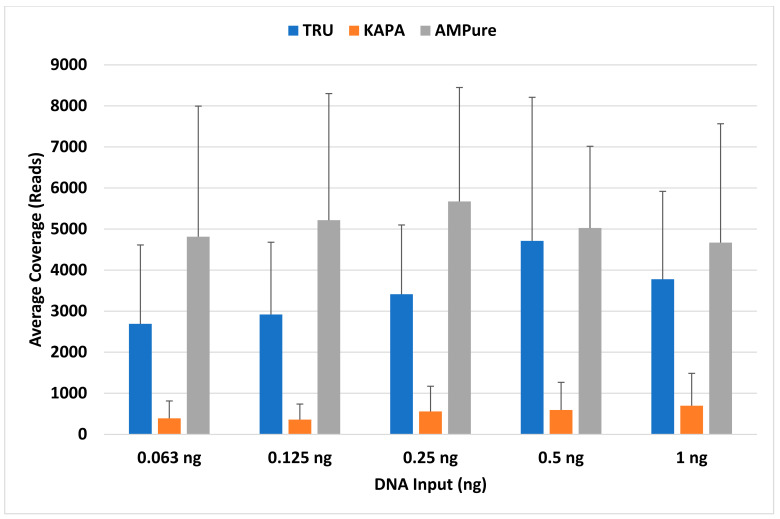
Average coverage on the *y* axis for each DNA input (0.063–1 ng) amount on the *x* axis for each library preparation/purification method: TruSeq library preparation with SPBs (blue), KAPA library preparation with KPBs (orange), and TruSeq library preparation with AMPure beads (gray). Error bars = ±SD.

For the TruSeq library preparation with AMPure XP beads, profile completeness ranged from 36.4% to 100%, with an average of 84% to 100% (Figure 6). Four male samples resulted in a profile completeness less than 100%. Three samples (one at 0.125 ng and two at 0.063 ng) were missing one allele (DYS389I). Additionally, a 1 ng sample was missing 42 out of 66 alleles (36.4% complete). This sample is likely a result of poor amplification efficiency, as the replicate resulted in 100% profile completeness. Overall, the TruSeq library preparation with AMPure XP purification resulted in the highest coverage across the three library preparation methods (Figure 7, gray bars). In contrast to the other two library preparation methods, 0.25 ng DNA input demonstrated the highest coverage, and was significantly higher (*p* = 0.004) than all but the 0.5 ng input (Figure 6). Coverage ranged from 103× (1 ng) to 22,320× (0.125 ng), with the 0.25 ng DNA input demonstrating the lowest and highest total coverage for a single concentration. The average coverage for these samples ranged from 4670× (1 ng) to 5673× (0.25 ng) (Figure 6). The locus that yielded the highest average coverage was DYS392, which was similarly observed in Silva et al. [35]. The locus that yielded the lowest average coverage, aside from DYS389I, was D3S1358 (Figure 3). Additionally, the heterozygote balance ranged from 0.40 (63 pg) to 1 (1 ng). The average HB was ≥0.70 for all DNA inputs, generally increasing as the DNA concentration increased, ranging from 0.70 (63 pg) to 0.85 (0.5 ng) (Supplemental Figure S2). All heterozygous loci had an average HB ≥ 0.65, with Amelogenin being the lowest and CSF1PO being the highest at 0.90 (Supplemental Figure S3), showing no heterozygote Imbalance. In contrast, Silva et al. [35] and Hölzl-Müller et al. [51] found that D2S1338 was the most susceptible locus to heterozygote imbalance out of all the autosomal loci in the 46GY panel. 

The locus coverage ranged from 0× (dropout) to a maximum coverage of 22,320×, with a dropout in 3 of 20 samples, totaling 17 instances of dropout (Figure 5, gray bars). Dropout occurred in 13 of 46 loci (28.2%) and only in male samples, with 15 instances in a 1 ng sample, and one instance in replicate samples at 0.063 ng. Additionally, no drop-in alleles were observed using TruSeq library preparation with AMPure XP purification.

However, there were 28 instances of below-threshold alleles in 2 out of 20 samples, both male, at 1 ng and 0.125 ng DNA inputs. A total of 21 of 46 loci (45.7%) resulted in two or fewer below-threshold alleles. The majority of below-threshold alleles (27 out of 28) were in the same 1 ng sample as the 15 instances of dropout, and it is believed that this was due to insufficient amplification (Figure 2, gray bars). All data were concordant with the reference samples, and negative controls were free of contamination.

### 3.4. Casework-Type Samples with the Enhanced Library Preparation Method

One objective for this study was to determine the most accurate library quantification kit. Of the three quantification methods tested, the PowerSeq^®^ Quant MS System provided the most accurate library quantification results prior to library normalization and sequencing. Because of this, we quantified our bone and hair libraries with the PowerSeq^®^ kit only. Library concentrations ranged from 1 nM to 20 nM for bone samples and <1 nM to 24 nM for hair samples (Supplemental Table S5). 

Of the six bone samples and four hair samples amplified in duplicate, one replicate set of hair samples (HQA and HQB) was contaminated and removed from analysis. Reagent blanks and negative controls were free of contamination. 

**Figure 8:**
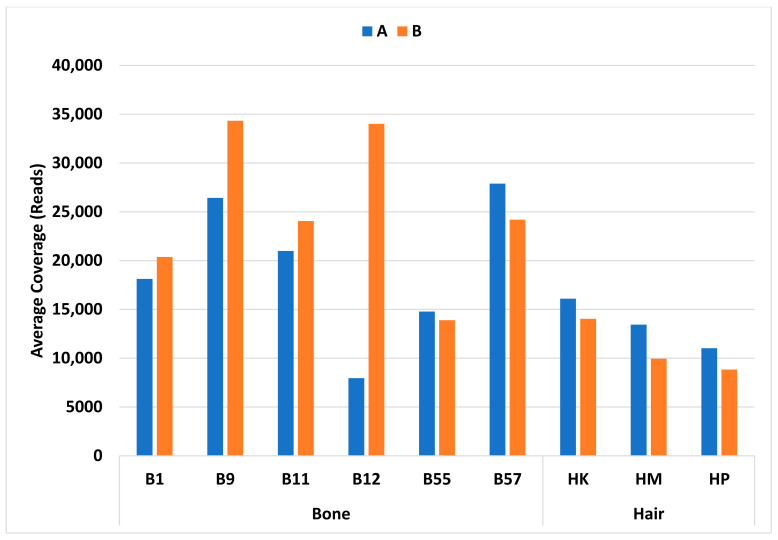
Average coverage on the *y* axis and bone or hair replicate samples on the *x* axis (Replicate A, blue; Replicate B, orange). These results represent the TruSeq library preparation kit using the AMPure XP beads.

All bone and hair samples resulted in 100% profile completeness and high sample coverage for all samples. Coverage ranged from 388× to 128,906× for bone samples and 130× to 56,077× for hair samples. The average coverage for bone samples ranged from 7955× (B12A) to 34,332× (B9B), and from 8835× (HPB) to 16,102× (HKA) for hair samples (Figure 8). In general, the coverage for hair samples was lower than that of bone samples. For all but two samples (B9 and B12), replicates resulted in similar coverage. For B9 replicate B the coverage was approximately 8000× higher than replicate A, and for B12 replicate B was approximately 26,000× higher than replicate A. This may be due to inefficient amplification, over dilution prior to end repair, stochastic variability, or inhibition from residual ethanol or cracked beads during cleanup. 

Additionally, the average HB was ≥0.74 for all samples, with bone and hair samples both yielding an overall average of ~0.80 (Supplemental Figure S7). All heterozygous loci had an average HB ≥ 0.67, with PentaE being the lowest and D1S1656 being the highest, at 0.89 (Supplemental Figure S8). Furthermore, PentaE was the only locus with an average HB below 0.70. 

All samples resulted in full profiles; therefore, no instances of dropout or below-threshold alleles were observed. Degradation Indices (DI) for these samples were between 0.78 and 2.57, with six samples indicating slight to moderate degradation (DI: 1–10) according to the Quantifiler Trio manual (Supplemental Table S1) [43]. However, the sequencing results displayed little to no indication of degradation.

Additionally, when MPS profiles were compared to CE reference profiles, between one and three isoalleles were identified in all but one donor (B12). Isoalleles were identified in five autosomal STRs, including D2S441 (x1), D5S818 (x1), D7S820 (x1), D13S317 (x4), and D21S11 (x2), and one Y-STR, DYS393 (x3). The five autosomal STRs listed above were five of the thirteen STRs found to have isometric heterozygosity in Hölzl-Müller et al. [51]. 

## 4. Conclusions

The goal of this research was to determine whether modifications to the 46GY workflow could improve the overall result quality and sequencing coverage beyond what is achieved following the manufacturer’s protocol, especially for casework-type samples. However, there was at least one limitation of this study, and that was small sample size. Only two buccal donors were used to assess the three library preparation methods prior to evaluating casework-type samples. In the first study, the 46GY panel was found to be compatible with the TruSeq DNA PCR-Free HT Library Preparation Kit and TruSeq SPBs, with very few instances of below-threshold alleles and no instances of dropout. Coverage was high, and profiles were nearly complete for all DNA inputs from 1 ng down to 63 pg with a heterozygote balance ≥ 0.70. Additionally, all three quantification methods tested (PowerSeq^®^ Quant MS System, KAPA Library Quantification Kit for Illumina^®^ Platforms, and KAPA using a data analysis template) overquantified libraries resulting in excessive dilution; however, the PowerSeq^®^ quantification system was the most accurate and provided usable quantification values that produced quality sequencing results. While there was compatibility between the 46GY panel and the KAPA HyperPrep Kit, this combination was not optimized for the best results. Results demonstrated low coverage and over 500 instances of dropout and below-threshold alleles, with heterozygote balance as low as 0.14 at 63 pg input. This also confirmed that the PowerSeq^®^ quantification kit was the most accurate prior to sequencing. Following the establishment of the more optimal library preparation kit (TruSeq), the next step was to determine if the use of AMPure XP beads in lieu of SPBs would improve downstream results. This method resulted in the highest overall coverage and heterozygote balance ≥ 0.70 at all DNA input amounts; however, 45 instances of dropout and below-threshold alleles were observed due to inefficient amplification. Lastly, bone and hair samples were tested with the most optimal library and quantification methods, as determined by the previous three studies, i.e., the TruSeq library preparation with SPBs and PowerSeq^®^ quantification, respectively. All casework-type samples resulted in full profiles with high coverage, no instances of dropout or below-threshold alleles, and a heterozygote balance ≥ 0.75. Overall, the 46GY panel produced the highest quality results with the manufacturer’s protocol. However, while not stated in the protocol, the authors recommend additional QC steps post-normalization for the best quality sequencing results. Quantifying the normalized library pool ensures that the correct concentrations of sequencing pool components are used to dilute and denature the pool prior to sequencing.

## Data Availability

All available data may be found in the manuscript and in Supplementary data files.

## Supplementary Materials

The following supporting information can be downloaded at: https://www.mdpi.com/article/10.3390/genes14050977/s1, Figure S1: Quantification of libraries prepared with the TruSeq library preparation kit using SPBs; Figure S2: Average heterozygote balance per library preparation/purification method; Figure S3: Average heterozygote balance locus per library preparation/purification method; Figure S4: Quantification of libraries prepared with the KAPA library preparation kit using KPBs; Figure S5: Average coverage per locus for KAPA library preparation with KPBs; Figure S6: Quantification of libraries prepared with the TruSeq library preparation kit using AMPure XP beads; Figure S7: Average heterozygote balance per bone or hair sample; Figure S8: Average heterozygote balance per locus for bone and hair samples; Table S1: Information regarding bone and hair samples used in Part 4 of this study; Table S2: TruSeq library prep using SPBs; Table S3: KAPA library prep using KPBs; Table S4: TruSeq library prep using AMPure XP; Table S5: Casework-type samples using TruSeq library prep and AMPure purification.

## References

[1] Wiegand P., Budowle B., Rand S., Brinkmann B. (1993). Forensic validation of the STR systems SE33 and TC11. Int. J. Leg. Med..

[2] Schmitt C., Schmultzler A., Prinz M., Staak M. (1994). High sensitive DNA typing approaches for the analysis of forensic evidence: Comparison of nested variable number of tandem repeats (VNTR) amplification and a short tandem repeats (STR) polymorphism. Forensic Sci. Int..

[3] Urquhart A., Kimpton C.P., Downes T.J., Gill P. (1994). Variation in short tandem repeat sequences—A survey of twelve microsatellite loci for use as forensic identification markers. Int. J. Leg. Med..

[4] FBI National Press Office The FBI’s Combined DNA Index System (CODIS) Hits Major Milestone. 21 May 2021. The FBI’s Combined DNA Index System (CODIS) Hits Major Milestone—FBI. https://www.fbi.gov/news/press-releases/the-fbis-combined-dna-index-system-codis-hits-major-milestone.

[5] Fordyce S.L., Ávila-Acros M., Rockenbauer E., Bǿrsting C., Frank-Hansen R., Petersen F.T., Willerslev E., Hansen A.J., Morling N., Gilbert M.T.P. (2011). High-throughput sequencing of core STR loci for forensic genetic investigations using the Roche Genome Sequencer FLX platform. Biotechniques.

[6] Bornman D.M., Hester M.E., Schuetter J.M., Kasoji M.D., Minard-Smith A., Barden C.A., Nelson S.C., Godbold G.D., Baker C.H., Yang B. (2012). Short-read, high-throughput sequencing technology for STR genotyping. Biotech. Rapid Dispatches.

[7] van der Gaag K.J., de Leeuw R.H., Hoogenboom J., Patel J., Storts D.R., Laros J.F.J., de Knijff P. (2016). Massively Parallel Sequencing of Short Tandem Repeats—Population data and mixture analysis results for the PowerSeq™ system. Forensic Sci. Int. Genet..

[8] Ganschow S., Silvery J., Tiemann C. (2019). Development of a multiplex forensic identity panel for massively parallel sequencing and its systematic optimization using design of experiments. Forensic Sci. Int. Genet..

[9] Ralf A., van Oven M., González D.M., de Knijff P., van der Beek K., Wootton S., Lagacé R., Kayser M. (2019). Forensic Y-SNP analysis beyond SNaPshot: High-resolution Y-chromosomal haplogrouping from low quality and quantity DNA using Ion AmpliSeq and targeted massively parallel sequencing. Forensic Sci. Int. Genet..

[10] Tillmar A., Grandell I., Montelius K. (2019). DNA identification of compromised samples with massive parallel sequencing. Forensic Sci. Res..

[11] de la Puente M., Phillips C., Xavier C., Amigo J., Carracedo A., Parson W., Larue M.V. (2020). Building a custom large-scale panel of novel microhaplotypes for forensic identification using MiSeq and Ion S5 massively parallel sequencing systems. Forensic Sci. Int. Genet..

[12] Holland M.M., McQuillan M.R., O’Hanlon K.A. (2011). Second generation sequencing allows for mtDNA mixture deconvolution and high resolution detection of heteroplasmy. Croat. Med. J..

[13] Strobl C., Eduardoff M., Bus M.M., Allen M., Parson W. (2018). Evaluation of the precision ID whole MtDNA genome panel for forensic analyses. Forensic Sci. Int. Genet..

[14] Van Neste C., Van Nieuwerburgh F., Van Hoofstat D., Deforce D. (2012). Forensic STR analysis using massive parallel sequencing. Forensic Sci. Int. Genet..

[15] De Barba M., Miquel C., Lobréaux S., Quenette P.Y., Swenson J.E., Taberlet P. (2017). High-throughput microsatellite genotyping in ecology: Improved accuracy, efficiency, standardization and success with low-quantity and degraded DNA. Mol. Ecol. Resour..

[16] Elwick K., Bus M.M., King J.L., Chang J., Hughes-Stamm S., Budowle B. (2019). Utility of the Ion S5 and MiSeq FGx sequencing platforms to characterize challenging human remains. Leg. Med..

[17] Bronner I.F., Quail M.A., Turner D.J., Swerdlow H. (2009). Improved Protocols for Illumina Sequencing. Curr. Protoc. Hum. Genet..

[18] Guo F., Yu J., Zhang L., Li J. (2017). Massively parallel sequencing of forensic STRs and SNPs using the Illumina^®^ ForenSeq™ DNA Signature Prep Kit on the MiSeq FGx™ Forensic Genomics System. Forensic Sci. Int. Genet..

[19] Mehta B., Venables S., Roffey R. (2018). Comparison between magnetic bead and qPCR library normalisation methods for forensic MPS genotyping. Int. J. Leg. Med..

[20] Calafell F., Anglada R., Bonet N., González-Ruiz M., Prats-Muñoz G., Rasal R., Lalueza-Fox C., Bertranpetit J., Malgosa A., Casals F. (2016). An assessment of a massively parallel sequencing approach for the identification of individuals from mass graves of the Spanish Civil War (1936–1939). Electrophoresis.

[21] Verogen Case Study How Next Generation Sequencing Resolved a Difficult Case, Leading to the First Criminal Conviction of its Kind. Verogen 2019. Pub. No. VD2019024. https://cdn2.hubspot.net/hubfs/6058606/Verogen-First-NGS-Court-Case-Study_Final_VD2019024_8.5x11-web.pdf.

[22] Brandhagen M.D., Just R.S., Irwin J.A. (2020). Validation of NGS for mitochondrial DNA casework at the FBI Laboratory. Forensic Sci. Int. Genet..

[23] Finaughty C., Reid K.M., Alli I.H., Heathfield L.J. (2020). A first for forensic genetics in Africa: Successful identification of skeletal remains from the marine environment using massively parallel sequencing. Forensic Sci. Int. Genet..

[24] Cuenca D., Battaglia J., Halsing M., Sheehan S. (2020). Mitochondrial Sequencing of Missing Persons DNA Casework by Implementing Thermo Fisher’s Precision ID mtDNA Whole Genome Assay. Genes.

[25] Gorden E.M., Sturk-Andreaggi K., Warnke-Sommer J., Hazelwood A., Barritt-Ross S., Marshall C. (2020). Next generation sequencing of STR artifacts produced from historical bone samples. Forensic Sci. Int. Genet..

[26] Ralf A., Kayser M. (2021). Investigative DNA analysis of two-person mixed crime scene trace in a murder case. Forensic Sci. Int. Genet..

[27] Bottino C., Silva R., Moura-Neto R.S. (2021). Resolving a human identification case for the Rio de Janeiro Police with massively parallel sequencing of mtDNA using a proposed pipeline. Genet. Mol. Res..

[28] Churchill J.D., Schmedes S.E., King J.L., Budowle B. (2016). Evaluation of the Illumina^®^ Beta Version ForenSeq™ DNA Signature Prep Kit for use in genetic profiling. Forensic Sci. Int. Genet..

[29] Xavier C., Parson W. (2017). Evaluation of the Illumina ForenSeq™ DNA Signature Prep Kit—MPS forensic application for the MiSeq FGx™ benchtop sequencer. Forensic Sci. Int. Genet..

[30] Wang Z., Zhou D., Wang H., Jia Z., Liu J., Qian X., Li C., Hou Y. (2017). Massively parallel sequencing of 32 forensic markers using the Precision ID GlobalFiler™ NGS STR Panel and the Ion PGM™ System. Forensic Sci. Int. Genet..

[31] Müller M., Alonso A., Barrio P.A., Berger B., Bodner M., Martin P., Parson W. (2018). Systematic evaluation of the early access applied biosystems precision ID Globalfiler mixture ID and Globalfiler NGS STR panels for the ion S5 system. Forensic Sci. Int. Genet..

[32] Tao R., Qi W., Chen C., Zhang J., Yang Z., Song W., Zhang S., Li C. (2019). Pilot study for forensic evaluations of the Precision ID GlobalFiler™ NGS STR Panel v2 with the Ion S5™ system. Forensic Sci. Int. Genet..

[33] Gettings K.B., Kiesler K.M., Faith S.A., Montano E., Baker C.H., Young B.A., Guerrieri R.A., Vallone P.M. (2016). Sequence variation of 22 autosomal STR loci detected by next generation sequencing. Forensic Sci. Int. Genet..

[34] Silva D.S.B.S., Sawitzki F.R., Scheible M.K.R., Bailey S.F., Alho C.S., Faith S.A. (2018). Genetic analysis of Southern Brazil subjects using the PowerSeq™ AUTO/Y system for short tandem repeat sequencing. Forensic Sci. Int. Genet..

[35] Silva D.S.B.S., Sawitzki F.R., Scheible M.K.R., Bailey S.F., Williams C.L., Allwood J.S., Just R.S., Schuetter J., Skomrock N., Minard-Smith A. (2020). Sequence-based autosomal STR characterizationin four US populations using PowerSeq™ Auto/Y system. Forensic Sci. Int. Genet..

[36] Moura-Neto R., King J.L., Mello I., Dias V., Crysup B., Woerner A.E., Budowle B., Silva R. (2021). Evaluation of Promega PowerSeq™ Auto/Y systems prototype on a admixed sample of Rio de Janeiro, Brazil: Population data, sensitivity, stutter and mixture studies. Forensic Sci. Int. Genet..

[37] Zeng X., King J.L., Stoljarova M., Warshauer D.H., LaRue B.L., Sajantila A., Patel J., Storts D.R., Budowle B. (2015). High sensitivity multiplex short tandem repeat loci analyses with massively parallel sequencing. Forensic Sci. Int. Genet..

[38] Hall C.L., Kesharwani R.K., Phillips N.R., Planz J.V., Sedlazeck F.J., Zascavage R.R. (2022). Accurate profiling of forensic autosomal STRs using Oxford Nanopore Technolgies MinION device. Forensic Sci. Int. Genet..

[39] Montano E.A., Bush J.M., Garver A.M., Larijani M.M., Wiechman S.M., Baker C.H., Wilson M.R., Guerrieri R.A., Benzinger E.A., Gehres D.N. (2018). Optimization of the Promega PowerSeq™ Auto/Y system for efficient integration within a forensic DNA laboratory. Forensic Sci. Int. Genet..

[40] FBI Laboratory Quality System Documents DNA-510.04. Procedure for the Semi-Automated Extraction of DNA. DNA-510-04 Procedure for the Semi-Automated Extraction of DNA.pdf—FBI Lab Vault. https://fbilabqsd.fbi.gov/file-repository/dna/quality-assurance.

[41] FBI Laboratory Quality System Documents BIO-513-00. Extraction of DNA from Hair and Keratinized Tissue. BIO-513-00 Extraction of DNA from Hair and Keratinized Tissue.pdf—FBI Lab Vault. https://fbilabqsd.fbi.gov/file-repository/dna/quality-assurance.

[42] FBI Laboratory Quality System Documents DNA-511-01. Procedures for Preparation and Extraction of Calcified Tissue Samples. DNA-511-01 Procedures for Preparation and Extraction of Calcified Tissue Samples.pdf—FBI Lab Vault. https://fbilabqsd.fbi.gov/file-repository/dna/quality-assurance.

[43] Applied Biosystems by Thermo Fisher Scientific Quantifiler™ HP and Trio DNA Quantification Kits User Guide. Publication 4485354, Revision G. Document Connect. thermofisher.com.

[44] Promega Corporation PowerSeq™ 46GY System Technical Manual. TM522 8/17. www.promega.com.

[45] Promega Corporation PowerSeq® 46GY System Technical Manual. TM522 Revised 3/22. www.promega.com.

[46] Promega Corporation PowerSeq™ Quant MS System Technical Manual. TM511 Revised 5/20. www.promega.com.

[47] KAPABIOSYSTEMS by Roche KAPA Library Quantification Kit Illumina® Platforms Technical Data Sheet. KR0405—v9.17. Sequencing.Roche.com/Support. https://rochesequencingstore.com/wp-content/uploads/2022/07/KAPA-Library-Quantification-Kit-Technical-Data-Sheet.pdf.

[48] Illumina Inc. Sequencing Analysis Viewer Software. 15020619 Rev F. https://support.illumina.com/content/dam/illumina-support/documents/documentation/software_documentation/sav/sequencing-analysis-viewer-user-guide-15020619-f.pdf.

[49] King J.L., Woerner A.E., Mandape S.N., Kapema K.B., Moura-Neto R.S., Silva R., Budowle B. (2021). STRait Razor Online: An enhanced user interface to facilitate interpretation of MPS data. Forensic Sci. Int. Genet..

[50] Scientific Working Group on DNA Analysis Methods (2019). Addendum to “SWGDAM Interpretation Guidelines for Autosomal STR Typing by Forensic DNA Testing Laboratories” to Address Next Generation Sequencing. https://www.swgdam.org/publications.

[51] Hölzl-Müller P., Bodner M., Berger B., Parson W. (2021). Exploring STR sequencing for forensic DNA intelligence databasing using the Austrian National DNA Database as an example. Int. J. Leg. Med..

[52] Moon M.H., Hong S.R., Shin K.J. (2022). Sequence variations of 31 Y-chromosomal short tandem repeats analyzed by massively parallel sequencing in three U.S. population groups and Korean population. J. Korean Med. Sci..

[53] Riman S., Iyer H., Borsuk L.A., Vallone P.M. (2020). Understanding the characteristics of sequence-based single-source DNA profiles. Forensic Sci. Int. Genet..

